# Small molecules related to adrenomedullin reduce tumor burden in a mouse model of colitis-associated colon cancer

**DOI:** 10.1038/s41598-017-17573-x

**Published:** 2017-12-13

**Authors:** Laura Ochoa-Callejero, Josune García-Sanmartín, Sonia Martínez-Herrero, Susana Rubio-Mediavilla, Judit Narro-Íñiguez, Alfredo Martínez

**Affiliations:** 1 Angiogenesis Group, Oncology Area, CIBIR, 26006 Logroño, Spain; 2 Pathology Service, Hospital San Pedro, 26006 Logroño, Spain

## Abstract

To investigate the contribution of adrenomedullin (AM) and its gene-related peptide, proadrenomedullin N-terminal 20 peptide (PAMP), to the progression and potential treatment of colon cancer we studied the effects of four small molecules (SM) related to AM and PAMP on a mouse model of colon cancer. For each SM, four experimental groups of male mice were used: (i) Control group; (ii) SM group; (iii) DSS group (injected with azoxymethane [AOM] and drank dextran sulfate sodium [DSS]); and (iv) DSS + SM group (treated with AOM, DSS, and the SM). None of the mice in groups i and ii developed tumors, whereas all mice in groups iii and iv developed colon neoplasias. No significant differences were found among mice treated with PAMP modulators (87877 and 106221). Mice that received the AM negative modulator, 16311, had worse colitis symptoms than their control counterparts, whereas mice injected with the AM positive modulator, 145425, had a lower number of tumors than their controls. SM 145425 regulated the expression of proliferation marker Lgr5 and had an impact on microbiota, preventing the DSS-elicited increase of the *Bacteroides/Prevotella* ratio. These results suggest that treatment with AM or with positive modulator SMs may represent a novel strategy for colon cancer.

## Introduction

Colorectal cancer (CRC) is the third most commonly diagnosed cancer worldwide. It is responsible for the death of about 200,000 people each year in Europe^1^ and it is expected to cause about 50,260 deaths and 135,430 new cases during 2017 in the United States^2^. Standard treatment consists on a combination of neoadjuvant chemoradiotherapy followed by surgery, but the response to this treatment and patient survival are heterogeneous^3^.

Adrenomedullin (AM) is a 52 amino acid peptide, in humans, that belongs to the calcitonin/calcitonin gene-related peptide family, which comprises also amylin and another peptide with high homology to AM, known as AM2 or intermedin^4,5^. AM is synthesized as part of a larger precursor molecule, termed preproadrenomedullin. This precursor consists of 185 amino acids in humans and contains a 21-amino acid N-terminal signal peptide that immediately precedes a 20-amino acid amidated peptide, designated proadrenomedullin N-terminal 20 peptide or PAMP. AM exerts its actions through a combination of the calcitonin receptor like receptor or CLR; and either receptor activity-modifying protein 2 (RAMP2) or RAMP3 (known as AM_1_ and AM_2_ receptors, respectively)^4^.

AM and PAMP are expressed throughout the gastrointestinal tract, being specially abundant in the neuroendocrine cells of the gastrointestinal mucosa; in the enterochromaffin-like and chief cells of the gastric fundus; and in the submucosa of the duodenum, ileum, and colon. This wide distribution in the gastrointestinal tract suggests that AM and PAMP may act as gut hormones regulating many physiological and pathological conditions^6^.

AM is widely expressed in a variety of tumor types^7^ and several studies have provided evidence that AM is involved in tumor initiation and progression^8^.

AM ligand and receptor overexpression in colonic cancers has been previously reported^9–13^ and a correlation between higher AM levels and lower disease-free survival has been described^10,14–16^. Furthermore, antibodies against either the peptide or the receptor reduce the growth of xenografted tumors^17,18^. Nevertheless, there is some controversy on whether AM is good or bad for colon cancer patients since application of the peptide clearly reduces inflammation and clinical severity in human and mouse models of colitis^19–21^, which is an important risk factor for colon cancer. In addition, AM has a protective role in gastrointestinal diseases^10,22^, and ameliorates the severity of these gut pathologies^15,23^.

Furthermore, AM and PAMP are antimicrobial peptides found in most epithelial surfaces and body secretions^24,25^ and their presence or absence modifies the composition of gut microbiota^20^.

Several pharmacological modulators of AM and PAMP have been described and they can be used to intervene in all physiological and pathological conditions where these peptides play a role. These modulators include monoclonal antibodies^26^, polyclonal antibodies against either the peptide^27,28^ or the receptors^17^, the peptide fragments AM22-52^29^ or PAMP12-20^30^, and small interfering RNAs^31^. In addition, several small molecules (SM) have been identified which can either increase or decrease AM or PAMP functions^30,32^.

To further investigate the contribution of AM and its gene-related peptide, PAMP, to the progression and potential treatment of colon cancer we have studied the effects of several SM related to AM and PAMP on a mouse model of colon cancer. Four SM were tested: 16311 (a negative modulator of AM), 145425 (a positive modulator of AM), 87877 (a negative modulator of PAMP), and 106221 (a positive modulator of PAMP)^30,32^.

## Results

### PAMP-related SMs do not modify cancer phenotype

PAMP modulators (87877 & 106221) did not modify colon cancer status or any of the other parameters tested (data not shown), indicating that PAMP may not play a major role in colon cancer or that the PAMP-related SMs were not very effective.

### SM 16311, but not 145425, increases weight loss and severity index in AOM-and DSS-treated mice

**Figure 1:**
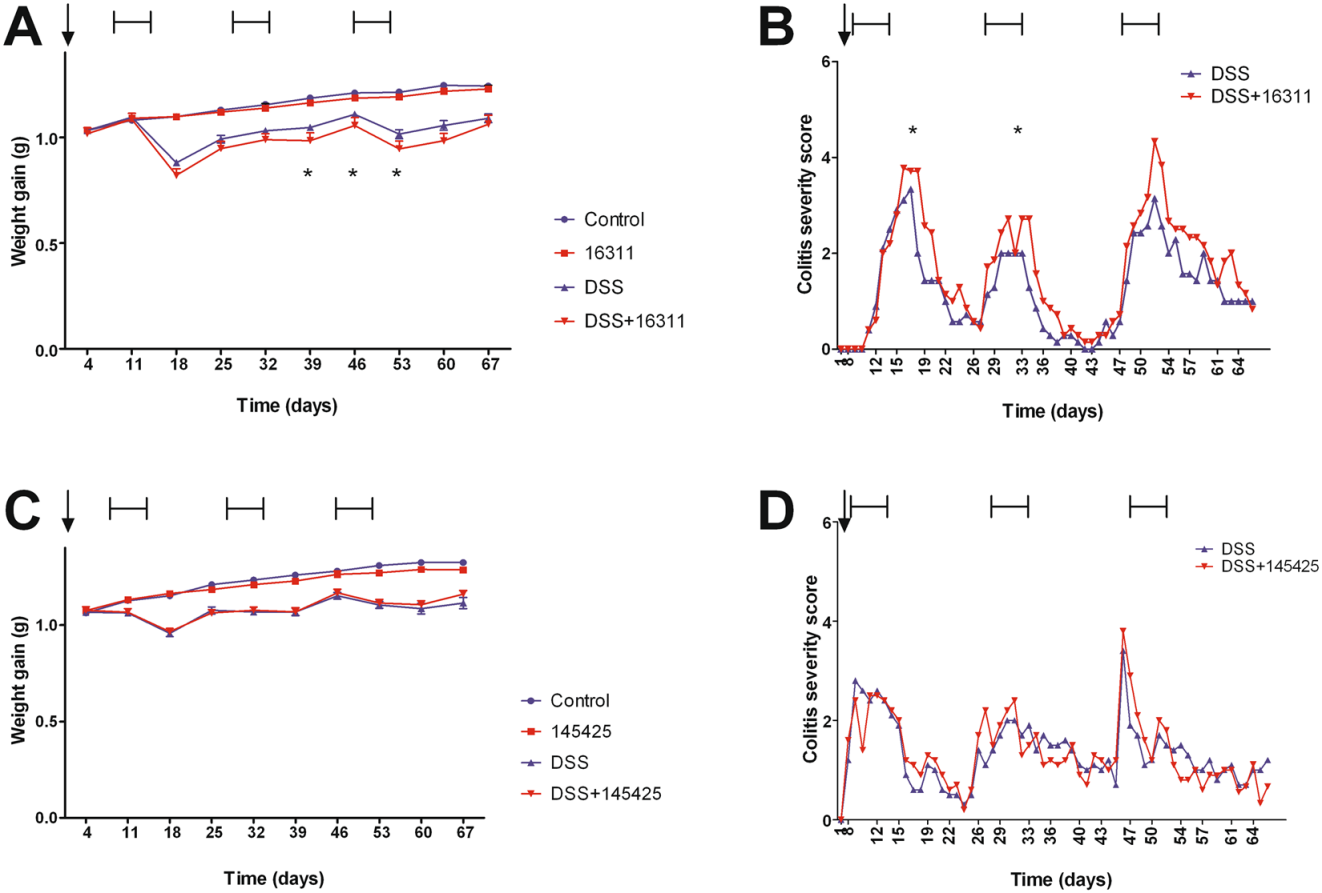
SM 16311 had a negative influence in the health status of DSS-treated mice. Body weight changes were recorded weekly in the four experimental groups treated with 16311 (**A**) or with 145425 (**C**) and are represented as weight gain. Mice treated with AOM and 3 cycles of DSS present weight loses following each cycle. SM 16311-treated mice experienced a significantly higher percentage of weight loss than their DSS counterparts. Colitis symptoms were scored on a 0–12 point scale in 16311 (**B**) and 145425 (**D**)-treated mice. Mice not receiving DSS had a 0 index at all times and are not shown. SM 16311-treated mice exhibited severe colitis symptoms reaching higher scores than their DSS-treated counterparts (**B**). SM 145425 had no effect on any of the parameters studied (**C**,**D**). Data are shown as mean ± SEM. ANOVA test. Asterisks represent time points at which statistically significant differences between 16311-treated and untreated mice exposed to DSS were found (Bonferroni); *p < 0.05.

The aim of this work was to analyze the impact of AM on colitis-associated CRC initiation and progression. This protocol allowed us to investigate the effect of SM modulators of AM on the severity of DSS-induced colitis. Control groups (Control and SM-treated) maintained body weight (Fig. 1A,C) and did not suffer major clinical symptoms (not shown). As expected, DSS treated mice experienced weight loss (18.3% over initial weight) (ANOVA+ Bonferroni p < 0.001) (Fig. 1A,C), and worse colitis symptoms such as dehydration, diarrhea, and rectal bleeding compared to control/untreated counterparts (Fig. 1B,D) (ANOVA + Bonferroni p < 0.001). In addition, mice that received DSS + 16311 experienced a significantly deeper weight loss (more than 20% over initial weight) (Fig. 1A), and more severe colitis symptoms (Fig. 1B) than mice treated with DSS. In contrast, 145425-treatment did not show any difference on these parameters over the DSS treatment (Fig. 1C,D).

### AM positive modulator, 145425, reduces tumor burden and colon weight/length ratio

**Figure 2:**
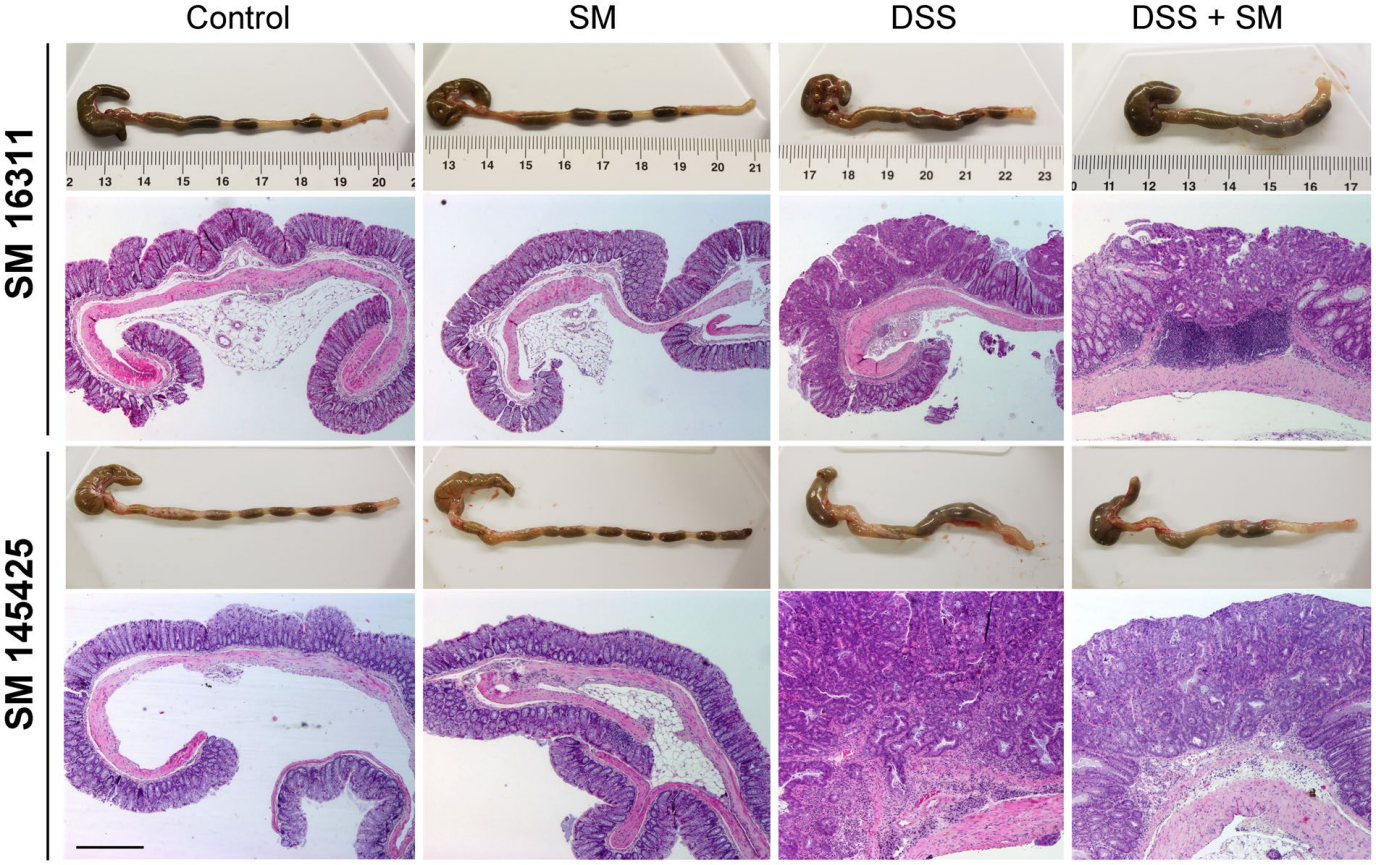
Effect of 16311 & 145425 on DSS-induced colon injury. Macroscopic aspect of the colon in the four experimental groups treated with either 16311 or 145425. The four experimental groups were: untreated (Control), SM-treated (SM), DSS-treated (DSS), SM and DSS-treated (DSS+SM). DSS treatment caused local inflammation on the colon. However, this inflammatory response was aggravated by 16311 and prevented in 145425-treated mice. Histological images were stained with hematoxylin-eosin. Histological analysis of untreated or SM-treated mice did not show any abnormality. The colon of the DSS group had numerous atypic cells and large areas occupied by polyps and tumors accompanied by lymphocyte infiltrates. After 16311 treatment some tumors invaded the submucosa (T1). When 145425 was present most tumors were classified as carcinoma *in situ* (Tis). Scale bar = 200 µm.

**Figure 3:**
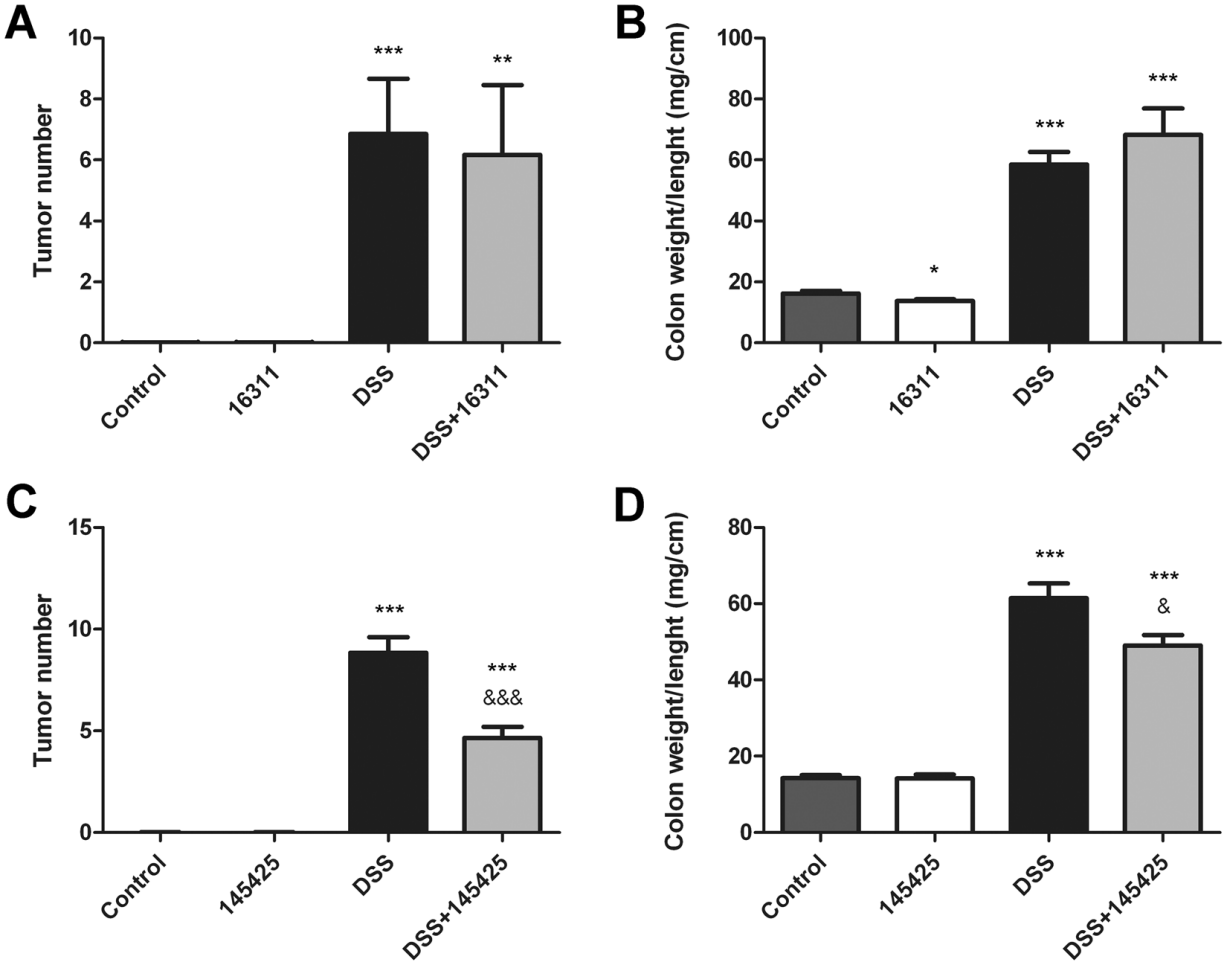
Number of tumors and relative weight of the colon. Number of tumors (**A**,**C**) and relative weight of the colon (**B**,**D**) was calculated in the four experimental groups. There is a significant increase in the number of tumors and weight/length ratio of the colon in all DSS-treated mice when compared with their respective controls. SM 145425 significantly reduced the number of tumors in treated mice (**C**). The reduction in the number of tumors was also evidenced by a reduction in colon weight (**D**). SM 16311 had no significant effect on any of these parameters (**A**,**B**). Data are shown as mean ± SEM. Kruskal‐Wallis test followed by Mann Whitney. Asterisks represent statistically significant differences with mice not receiving DSS; *p < 0.05; **p < 0.01; ***p < 0.001. Ampersands indicate statistically significant differences between DSS treated mice; ^&^p < 0.05; ^&&&^p < 0.001.

At the macroscopic level, untreated animals present the characteristic size and oval shape of mice feces (Fig. 2). Treatment with the SMs had no effects on feces or intestinal morphology (Fig. 2). However, DSS administration caused gut pathology characterized by more liquid feces, macroscopic inflammation, and colonic thickening. SM 16311 worsened (Fig. 2) but 145425 improved (Fig. 2) the macroscopic appearance of the colon in comparison with DSS-treated animals. All mice in DSS-treated groups (DSS and DSS+SM) developed colon neoplasias that were classified either as adenomas, carcinomas *in situ*, or adenocarcinomas (Table 1). Number of tumors (Fig. 3A,C) and the colon weight/length ratio (Fig. 3B,D) was recorded in the four groups. None of the mice belonging to control groups (Control and SM) developed any tumor or had any pathological finding, indicating that the SMs do not present overt toxicity. As expected, treatment with DSS resulted in a significant increase on the number of tumors (p < 0.001) and on the colon weight/length ratio (p < 0.001). SM 16311 had no effect on these parameters (Fig. 3A,B) whereas 145425 significantly reduced the number of tumors (p < 0.001) (Fig. 3C) and the colon weight/length ratio (p < 0.05) (Fig. 3D).

**Table 1:** Pathological data for adenocarcinomas of the colon.

Groups	Grade		
	T0	Tis	T1
DSS	28,6	71,4	0
DSS + 16311	0	71,4	28,6
DSS	0	88,9	11,1
DSS + 145425	0	100	0

Data are presented as percentage of tumors for each experiment. T0 = No evidence of primary tumor; Tis = Carcinoma *in situ*, invasion of lamina propria/muscularis mucosae; T1= Tumor invades submucosa. Fisher’s exact test indicated that there are no significant differences between treatments.

### SMs do not modify the expression of inflammatory cytokines in the colon

In an attempt to identify the potential mechanism of AM modulators in the immune response, we examined gene expression patterns of different inflammatory cytokines. In DSS-treated mice, there was a significant increase in IFN-γ, TNF-α, IL-6, and IL-10 levels compared with their sham groups. The levels of these cytokines were not significantly affected by SM treatment (Supplementary Figure S1A–H).

### SMs modulate the expression of AM and AM2 in the colon

We also measured the mRNA expression levels of AM and AM2 in colon samples from all the experimental groups. SM 16311 significantly decreased the levels of AM in DSS-treated samples but had no influence on AM2 expression (Supplementary Figure S2A-B). Conversely, SM 145425 significantly elevated the levels of AM in DSS-treated tissues (Supplementary Figure S2C). Concerning AM2 expression, SM 145425 reduced AM2 levels in untreated animals but had no significant effects on DSS-treated mice (Supplementary Figure S2D).

### SM 145425 prevents DSS-related changes in Lgr5 and Erbb2 expression

**Figure 4:**
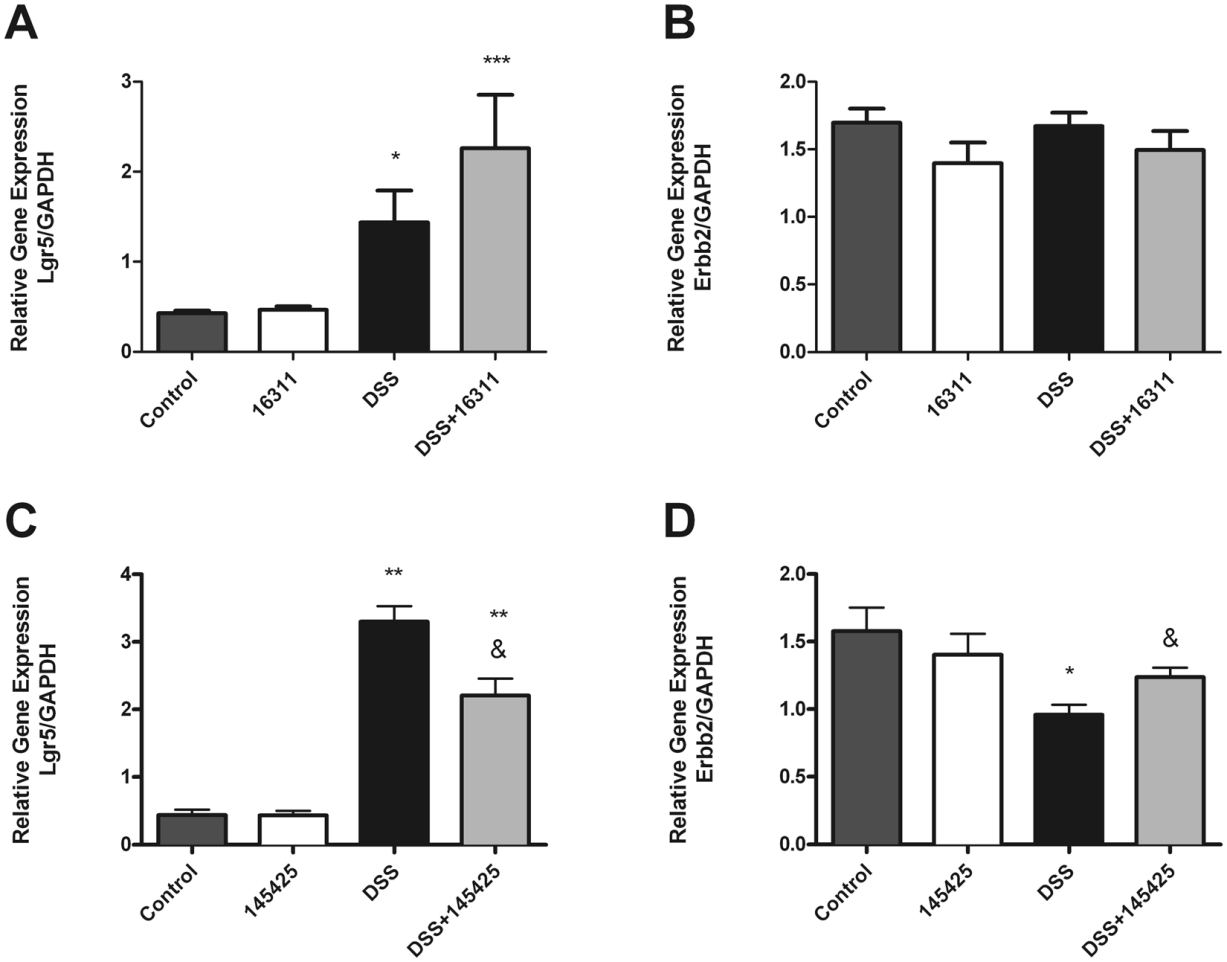
Gene expression in the colon. Stem cell marker Lgr5 significantly increased with DSS treatment (**A,C**), indicating extensive proliferation. This marker was significantly reduced by treatment with 145425 (**C**). On the other hand, Erbb2 had the reverse behavior, decreasing by DSS and being modulated by 145425 (**D**). Moreover, treatment with 16311 did not affect any of these two genes studied when compared with their respective DSS-treated controls (**A**,**B**). Data are shown as mean ± SEM. Kruskal‐Wallis test followed by Mann Whitney; *p < 0.05; **p < 0.01; ***p < 0.001. Ampersands indicate statistically significant differences between DSS treated mice; ^&^p < 0.05.

In DSS-treated mice, there was an increase in Lgr5 and a decrease in Erbb2 gene expression levels compared with their sham groups (Fig. 4A). SM 16311 did not significantly modify the expression of either Lgr5 or Erbb2 (Fig. 4A,B). A significant decrease of Lgr5 and a significant increase of Erbb2 was induced by 145425 administration in DSS-treated mice (p < 0.05) (Fig. 4C,D). Since 145425 seems to offer a therapeutic potential, we further investigated the influence of this SM in histopathology and microbiota modulation.

### SM 145425 reduces tumor growth in DSS treated mice

**Figure 5:**
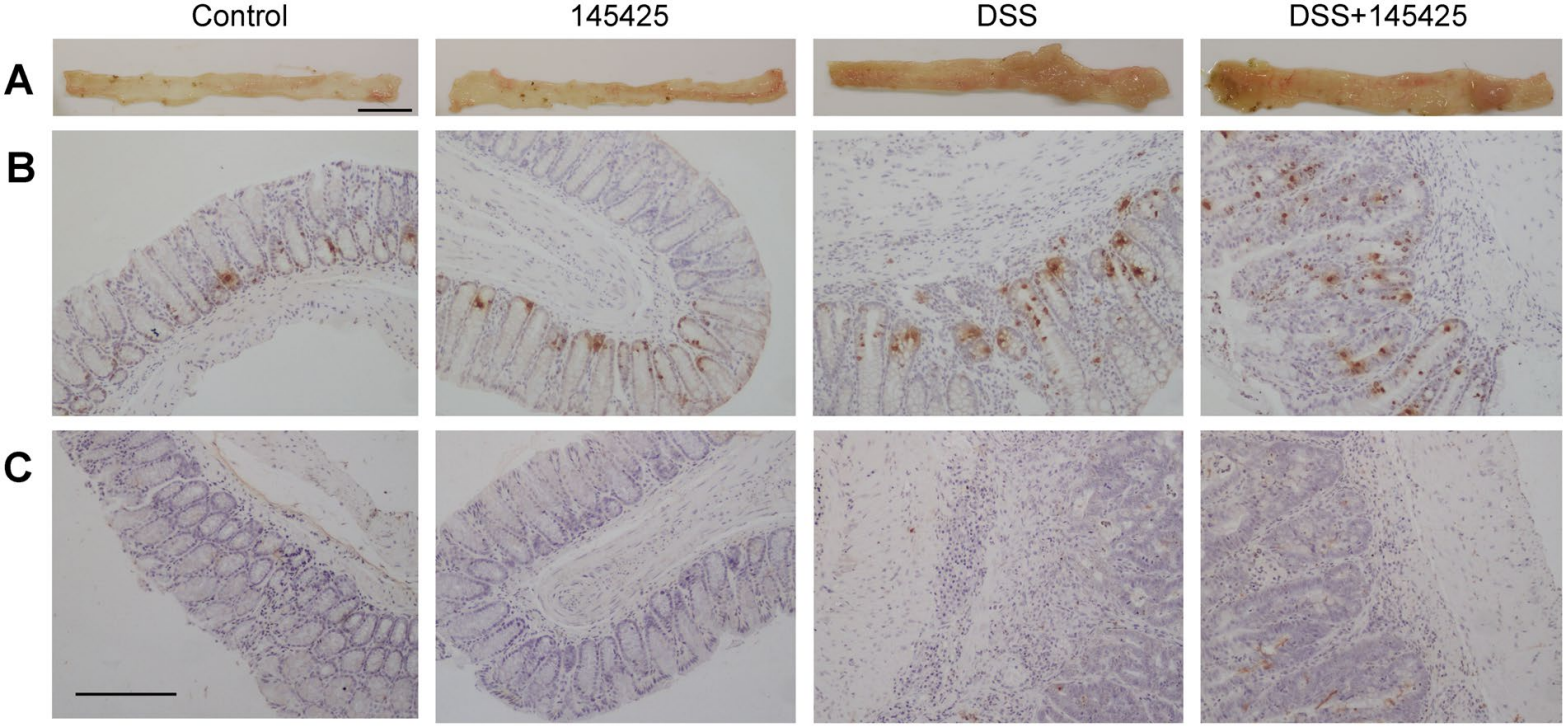
Morphological aspect of the mucosa in the four experimental groups. Representative macroscopic (**A**) and histological (**B**,**C**) appearance of the colon of the four experimental groups. The first 2 groups, Control and SM, displayed a normal colon morphology. The colon of the DSS group had numerous frank tumors. Treatment with 145425 reduced the number of tumors. Histological sections were stained with the proliferation marker anti Phospho-Histone H3 (PH3) antibody (**B**), and the TUNEL technique (**C**) to determine levels of proliferation and apoptosis, respectively. The number of proliferating cells was low in control animals whereas this number greatly increased in the DSS group. The number of proliferating cells was similar between DSS and DSS+SM groups (**B**). The TUNEL technique detected few apoptotic cells in the colon of animals belonging to control groups but the number increased in animals treated with DSS but did not vary after treatment with 145425 (**C**). Scale bar for A = 10 mm; for B and C = 100 µm.

Macroscopic inspection of colon and rectum provided evidence of different degrees of colonic inflammation and tumor burden in DSS-treated animals. Mice that were not exposed to DSS had a healthy mucosa (Fig. 5A), whereas DSS treatment resulted in a thickening of the mucosa and the presence of numerous polyps and tumors (Fig. 5A). Treatment with 145425 reduced the number of tumoral structures in the colon (Fig. 5A). In addition, a low number of proliferating (Fig. 5B) and apoptotic (Fig. 5C) cells were detected in control animals but this numbers greatly increased in the DSS groups. The number of proliferating and apoptotic cells was similar between DSS and DSS+SM groups. Moreover, myeloperoxidase activity was not affected by SM treatment (Supplementary Figure S3).

### Metagenomic analysis of gut bacterial populations

**Figure 6:**
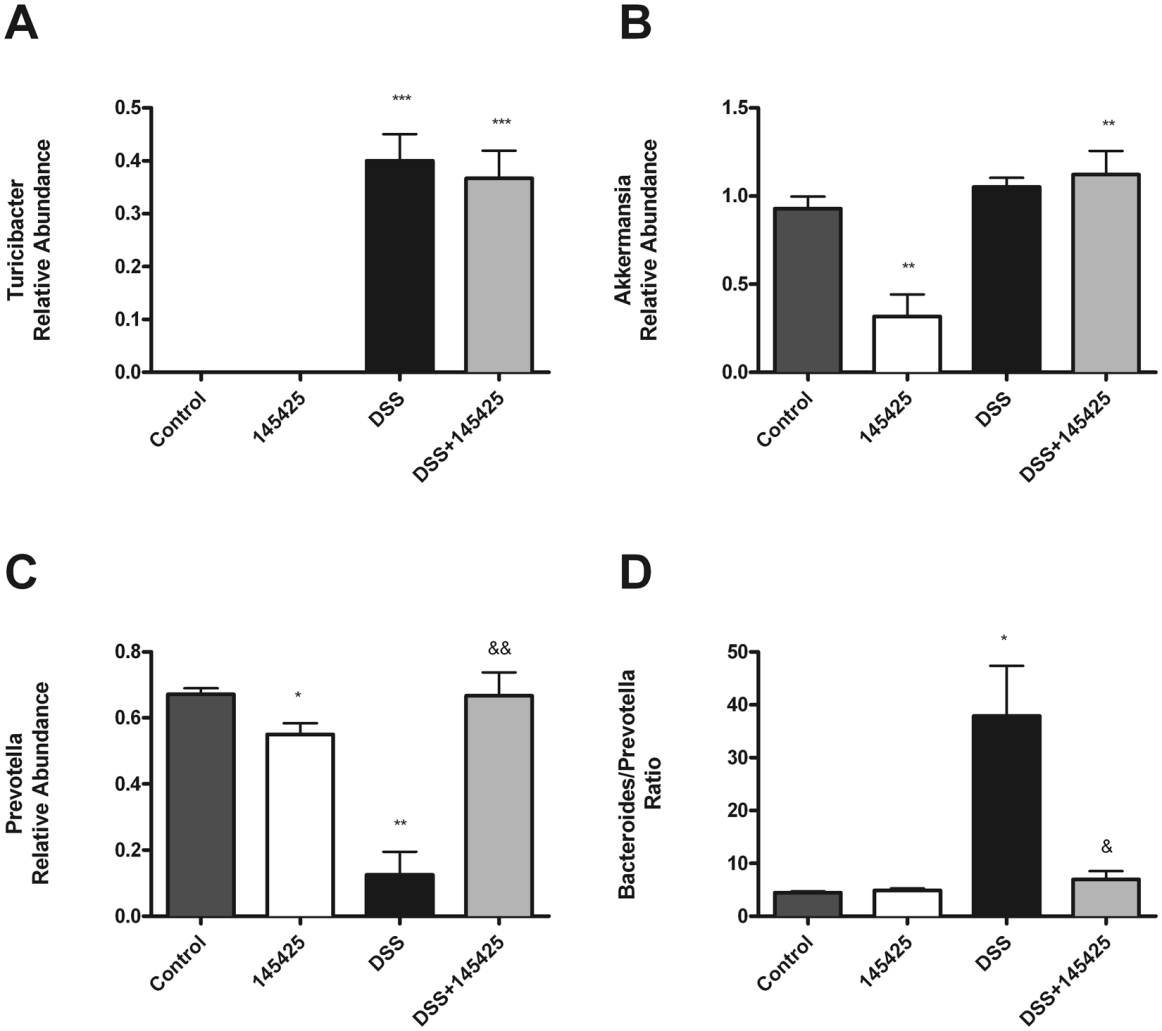
Relative abundance of particular microbiota genera in the four experimental groups. Genus *Turicibacter* increased significantly with DSS treatment (**A**). *Akkermansia* was significantly reduced by 145425 in untreated mice but that difference did not persist after DSS treatment (**B**). Interestingly, *Prevotella* decreased with DSS treatment but this decrease was completely prevented by 145425 (**C**). Besides, 145425 prevented the large increase in the *Bacteroides/Prevotella* ratio elicited by DSS (**D**). Data are shown as mean ± SEM. Kruskal‐Wallis test followed by Mann Whitney. Asterisks represent statistically significant differences with control; *p < 0.05; **p < 0.01; ***p < 0.001. Ampersands indicate statistically significant differences between DSS treated mice; ^&^p < 0.05; ^&&^p < 0.01.

Okayasu *et al*.^33^, among others, suggested a contribution of colonic bacteria or their products to the development of DSS-induced colitis. They observed increased numbers of *Enterobacteriaceae*, *Bacteroidaceae*, and *Clostridium* spp. in the colons of DSS-treated mice^33^. Using the same model we have studied the effects of AM modulator, 145425, on gut microbiota. Supplementary Figure S4 shows the relative abundance of the major bacterial phyla present in the gut in the four experimental groups. As expected, around 90% of the bacteria detected (93.4 and 94.4% for control and 145425 animals respectively) belong to the *Bacteroidetes* and *Firmicutes* phyla (Table 2). No significant differences were observed in major phyla elicited by either DSS treatement or the SM (Table 2). However, significant changes were observed in less represented phyla (Table 2). Thus, a significant reduction in the abundance of *Cyanobacteria and Tenericutes* was observed in DSS-treated mice (DSS and DSS+145425 groups) when compared with Control groups (Control and 145425). Surprisingly, the abundance of *Verrucomicrobia* was decreased in the 145425 group when compared to the other groups (Table 2). At the family level, DSS treatment resulted in a significant increase in the abundance of *Bacteroidaceae*, *Turicibacteraceae*, *Peptostreptococcaceae*, *Erysipelotrichaceae*, and *Alcaligenaceae* in comparison to control groups (Supplementary Table S2). In contrast, DSS treatment resulted in a significant decrease in the abundance of *Prevotellaceae*, *Paraprevotellaceae*, *Cyanobacteria;c__4C0d-2;o__YS2*, *Alphaproteobacteria*, and *Anaeroplasmataceae* (Supplementary Table S2). However, after 145425-treatment this decrease in the abundance of the *Prevotellaceae*, and *Paraprevotellaceae* was normalized (Supplementary Table S2,S3,S4). Interestingly, *Erysipelotrichaceae* was significantly increased and *Paraprevotellaceae* decreased by 145425. *Verrucomicrobiaceae* was significantly reduced by 145425 in untreated mice but that difference did not persist after DSS treatment (Supplementary Table S4). Concerning lower taxonomic levels (genus), DSS-treated mice exhibited a significant increase in the abundance of *Bacteroidales*, *Bacteroides, Turicibacter* (Fig. 6A), *Peptostreptococcaceae, Erysipelotrichaceae, Allobaculum and Sutterella* and a significant decrease in the presence of *Prevotella* (Fig. 6C), *Cyanobacteria;c__4C0d-2;o__YS2, Dorea, Alphaproteobacteria;o__RF32*, *and Anaeroplasma*. In some genera, SM 145425 was able to influence bacterial abundance in control animals. For instance, *Akkermansia* was significantly reduced by 145425 in untreated mice but that difference did not persist after DSS treatment (Fig. 6B). The case of *Prevotella* was particularly interesting: DSS greatly reduced *Prevotella* abundance but after 145425-treatment the presence of *Prevotella* was completely normalized (Fig. 6C). The ratio *Bacteroides/Prevotella* has been shown to have clinical relevance^34,35^. DSS induced a large increase of this ratio whereas 145425-treatment prevented this elevation completely (Fig. 6D). Supplementary Figure S5 shows the alpha and beta-diversity analysis.

**Table 2:** Relative abundance of the most representative phyla in the gut of Control, 145425, DSS and DSS-145425 groups.

	Taxonomic group (relative abundance [%])	Control	145425	DSS	DSS+145425
Phyla	*Firmicutes*	52.7 ± 3.2	57.1 ± 5.1	56.9 ± 5.6	53.1 ± 4.5
	*Bacteroidetes*	40.7 ± 2.6	37.3 ± 4.7	37.1 ± 5.4	41 ± 4.1
	*Cyanobacteria*	0.7 ± 0.1	0.7 ± 0.2	**0.4 ± 0.2	**0.3 ± 0.1
	*Tenericutes*	0.6 ± 0.2	0.7 ± 0.2	**0.2 ± 0.2	**0.2 ± 0.4
	*Verrucomicrobia*	0.9 ± 0.1	***0.3 ± 0.4	1.1 ± 0.1	1.1 ± 0.3
Ratio	*Bacteroidetes/Firmicutes*	0.78 ± 0.1	0.56 ± 0.3	0.58 ± 0.3	0.71 ± 0.3

Data are presented as mean values ± SEM. Kruskal‐Wallis test **p < 0.01 versus Control; ***p < 0.001 versus Control. Statistically significant differences are indicated by bold type.

## Discussion

In this study we have shown that AM positive modulation by 145425 treatment reduces tumor incidence in a mouse model of colitis-associated colon cancer and results in gut microbiota changes, thus linking AM with colorectal cancer initiation and progression. Previous studies have shown a strong positive effect using AM as treatment for colitis symptoms in rodents^22^ and humans^15,23^. Besides, this study shows that AM negative modulation by 16311 exacerbates colitis symptoms suggesting that AM prevents the symptoms of the disease in line with previous experiments^20,36^. Microscopic analysis of colonic sections confirmed data from macroscopic observations reflecting a tumor reduction in 145425-treated mice. These results demonstrated that AM exerts a protective action in DSS-induced experimental colitis, and are in line with previous papers^22^. As previously described^37^, three cycles of DSS result in the development of colitis-associated dysplasias and adenocarcinomas in approximately 15%–20% of mice which is in agreement with our data. No differences were found among mice treated with PAMP modulators (87877 and 106221), indicating that PAMP may not play a major role in colon cancer or that the PAMP-related SMs were not very effective.

Our data suggest that AM is a beneficial factor since this peptide significantly delays CRC development. This observation seems to be in contrast with previous studies where inhibition of AM or its receptors produced tumor reduction^17,18^. Previous experimental studies consisted on xenograft models whereas our working paradigm uses a long-term orthotopic carcinogen-induced model of CRC, which therefore is closer to the natural occurrence in humans and its results may be more relevant. In addition, our experimental model is very dependent on colitis as the trigger of CRC. Since AM has been shown repeatedly to be very efficient in reducing colitis symptoms^19–21^, the observed effects on CRC may be just a consequence of the milder colitis suffered by SM treated animals. Future studies should address whether AM is also protective in other models of colon cancer.

The DSS-induced colitis model induces high amounts of Th1 cytokines (TNF-α, IL-6)^22^. These cytokines exert potent proinflammatory effects that, when uncontrolled, can lead to tissue injury. Our results demonstrated that DSS administration induces an inflammatory response in the colon. However, SM treatment did not modify this response suggesting that these SMs play no role in immunoregulation.

Although the mechanism of action of the SMs implicates their direct binding to the mature peptide and a consequent modification of the peptide’s functions^30,32^, we also looked at the expression of AM and AM2 genes and their potential modulation by the SMs. As expected, there was no significant modification of AM levels in untreated animals but, in DSS-treated mice, there was a downregulation by the inhibitory SM (16311) and a significant upregulation by the positive modulator (145425), which would help to explain the beneficial effects of SM 145425 and the increase of colitis symptoms by SM 16311. The expression of AM2 was not modified by SM 16311 but SM 145425 reduced the expression of AM2 on untreated animals. This effect could be explained by an increase on the physiological actions of AM that, through an inhibitory feedback loop, may result in a reduction of AM2 expression.

The stem cell marker Lgr5 significantly increased with DSS treatment, indicating extensive proliferation. This marker’s increase was significantly prevented by treatment with 145425. Lgr5 is a marker of adult stem cells involved in self-renewal and cancer^38^. On the other hand, Erbb2 had the reverse behavior, decreasing by DSS and being modulated by 145425. Erbb2 regulates recovery from DSS-induced colitis by promoting mouse colon epithelial cell survival^39^. These results suggest that AM plays important cytoprotective and reparative roles in the colonic epithelium following injury, by promoting colon epithelial cell survival.

Another possible mechanism by which AM protects from colon cancer development is microbiota modulation. In a previous study we showed that mice lacking AM had an altered gut microbiota^20^. In this study, *Turicibacter* and *Bacteroidaceae* increased significantly with DSS treatment, in agreement with previous reports by Berry^40^ and Okayasu^33^, respectively. Interestingly, we observed that the positive modulator of AM (145425) induced a decrease in the *Bacteroides/Prevotella* ratio. This ratio is higher in colon cancer patients than in healthy controls^34,35^ and a greater abundance of *Prevotella* has been found in healthy rats than in CRC rats^41^. Furthermore, individuals consuming healthy diets have a lower *Bacteroides/Prevotella* ratio than people on high-calory diets^42,43^. Thus, we can hypothesize that the beneficial effects of 145425 may be partially mediated through this mechanism. Moreover, 145425 modulates some bacteria by itself, for example *Akkermansia* was significantly reduced by 145425 in untreated mice but that difference did not persists after DSS treatment. *Akkermansia* was more abundant in CRC samples^35^ which can represent an advantage for the 145425 treatment. Therefore, AM positive modulation is clearly associated with healthy changes in microbiota composition. The dysbiosis produced by DSS predisposes mice to worse colitis symptoms and 145425 prevents such microbiota modifications.

In conclusion, AM may have a protective role during the progression phase of colon cancer, and treatment with AM or with positive modulator SMs may represent a novel treatment for colon cancer.

## Material and Methods

### Colitis-associated cancer induction

The protocol was performed as previously described^44^. Briefly, treated animals received a single intraperitoneal (i.p) injection (10 mg/Kg) of the carcinogen azoxymethane (AOM) (Sigma-Aldrich, Madrid, Spain). One week later, animals were given 2.5% dextran sulfate sodium (DSS) (Sigma-Aldrich) in the drinking water for 1 week followed by 2 weeks of tap water. The DSS treatment was repeated for 2 additional cycles and tumorigenesis was examined 2 weeks after the last cycle. Untreated control mice received a saline injection instead of AOM and drank tap water only. Four SM were tested: 16311 (a negative modulator of AM), 145425 (a positive modulator of AM), 87877 (a negative modulator of PAMP), and 106221 (a positive modulator of PAMP). Sixty 8-week old male C57BL/6 mice were used in this study for each SM. Experimental groups were formed as follows: (i) Control group (injected i.p. with vehicle and drank regular water, n = 10); (ii) SM group (vehicle, regular water, and injected i.p. with the SM 3 times a week at a concentration of 20 nm/Kg, n = 10); (iii) DSS group (injected i.p. with AOM and drank DSS, n = 20); and (iv) DSS + SM group (treated with AOM, DSS, and the SM, n = 20). Small molecules were generously provided by the NCI Developmental Therapeutic Program (Frederick, MD), and their selection and characterization has been previously published^30,32^. All procedures involving animals were carried out in accordance with the European Communities Council Directive (2010/63/UE) and Spanish legislation (RD53/2013) on animal experiments and with approval from the ethical committee on animal welfare of our institution (Órgano Encargado del Bienestar Animal del Centro de Investigación Biomédica de La Rioja, OEBA-CIBIR).

### Clinical assessment of colitis

Mice were observed and weighed weekly. Assessments of rectal bleeding, diarrhea, prolapse, inactivity, and percent weight loss relative to baseline were scored according to the system described by Gommeaux *et al*.^45^ and used as a surrogate measure of colitis severity.

### Mouse sacrifice, macroscopic analysis, and tissue harvesting

All mice were sacrificed by an overdose of anesthesia (ketamine-xylazine) 70 days after AOM injection. Entire colons were dissected, rinsed with ice-cold phosphate buffer solution (PBS) to remove fecal residues, and weighed. Photographs of colon samples were taken using an *EOS50D* camera (Canon, Tokyo, Japan). Colon fragments were snap-frozen in liquid N_2_ and stored at −80 °C for further analysis. Central portions of colonic tissue were fixed in 10% buffered formalin

### Hematoxylin-eosin staining

Following fixation, tissues were dehydrated and paraffin embedded. Tissue sections (3 µm-thick) were rehydrated and stained with hematoxylin-eosin. Three sections from different colon pieces were analyzed for each animal and 3 random pictures were taken from each section with the 4x objective. At least 7 animals per group were included in the analysis.

### TUNEL staining

Colonic cells undergoing apoptosis were identified by means of a TUNEL assay kit (Promega, Madison, WI), following manufacturer’s instructions. Random pictures were taken from each section with the 10x objective.

### Immunohistochemical staining

Paraffin-embedded sections were rehydrated, and antigen retrieval was performed by heating in citrate buffer (pH 6.0) for 20 min at 96 °C. After blocking with normal donkey serum, sections were incubated overnight with Phospho-Histone H3 primary antibody (Cell Signaling, Danvers, MA) at 1:100. The next day, following several washes in PBS, a biotinylated donkey anti-rabbit (Jackson Immunoresearch, Suffolk, UK) at 1:500 was added for 60 min, followed by the ABC complex (Vector, Burlingame, CA) and developed with diaminobenzidine (Dako, Carpinteria, CA). Slides were counterstained with hematoxylin. Pictures were taken from each section with the 10x objective.

### RNA isolation and quantitative real-time PCR

RNA isolation, cDNA synthesis, and qRT-PCR were performed as described^46^. Briefly, total RNA was isolated from distal colon fragments using Qiagen RNeasy MiniKit (Qiagen, Hilden, Germany) with DNAse digestion step performed (Qiagen) according to manufacturer’s instructions. Total RNA (1 µg) of each sample was reverse transcribed using the SuperScriptR III Reverse Transcriptase Kit (Thermo Fisher Scientific, Waltham, MA). The synthesized cDNA was amplified by qRT-PCR with a 7300 real-time PCR System (Applied Biosystems, Foster City, CA) and gene expression was calculated using relative quantification by interpolation into a standard curve using RQ software (Applied Biosystems), as described^47^. All values were divided by the expression of the house keeping gene, GAPDH, to avoid potential loading errors. Target genes (IFN-γ, TNF-α, IL-6, IL-10, Lgr5, and Erbb2) and primers are described in Table S1 of Supplementary Material.

### Feces collection and DNA extraction

Fresh fecal contents were collected from each animal and weighed. DNA was subsequently extracted from fecal microbiota using the DNeasy Blood & Tissue Kit (Qiagen, Venlo, Netherlands). DNA purity and concentration were determined by a Nanodrop spectrophotometer (ND-1000; Thermo Fisher Scientific).

### Bacterial 16S rDNA massive sequencing and sequence postprocessing

Samples were amplified for the 16 S rRNA hypervariable sequences V3-V4 using Illumina recommended primers in a MiSeq Instrument (2 × 300 bp reads) (Illumina, INC, SanDiego, CA). Quality of sequenced reads was assessed by FastQC software (http://www.bioinformatics.babraham.ac.uk/projects/fastqc/). Raw reads were quality trimmed with Trimmomatic^48^. Reads were assigned into OTU categories with Qiime software^49^ by following the “pick open reference otus” methodology with Usearch61 clustering algorithm (http://www.ddrive5.com/usearch/). Taxonomic classification was performed by using the GreenGenes database^50^ at 97% of nucleotide identity. OTUs that were present at less than 0,01% of the total read counts on a per-sample basis were removed (spurious sequences). Raw normalization counts in the OTU table were normalized by the Cumulative Sum Scalling (CSS) methodology with metagenomeSeq R package (http://bioconductor.jp/packages/2.14/bioc/vignettes/metagenomeSeq/inst/doc/metagenomeSeq.pdf).

### Statistical analysis

All data sets were analyzed for normality and homoscedasticity. Normal data were analyzed by Unpaired Student’s t test or by 2-way ANOVA followed by Bonferroni post-hoc test. Data that did not follow a normal distribution were compared by Kruskal-Wallis test followed by Mann Whitney post-hoc test. For tumor grade comparisons the Fisher’s exact test was used. Analyses were performed with GraphPad Prism version 5.02 (GraphPad Software, Inc. La Jolla, CA). A p value < 0.05 was considered statistically significant.

## Electronic supplementary material

Supplementary material
