# Supplementary material for: Small molecules related to adrenomedullin reduce tumor burden in a mouse model of colitis-associated colon cancer
Source: Sci Rep. 2017 Dec 13;7:17488. doi: 10.1038/s41598-017-17573-x (PMC5727507; doi:10.1038/s41598-017-17573-x)
Supplement: Supplementary file 1 — Supplementary material [file 41598_2017_17573_MOESM1_ESM.doc]

**Supplementary material:**

Small molecules related to adrenomedullin reduce tumor burden in a mouse model of colitis-associated colon cancer

Laura Ochoa-Callejero, Josune García-Sanmartín, Sonia Martínez-Herrero, Susana Rubio-Mediavilla, Judit Narro-Íñiguez, Alfredo Martínez

**Supplementary Figure S1. Effects of 16311 and 145425 on mucosal inflammatory responses during colitis caused by DSS administration.** Expression of the principal pro‐ and anti‐inflammatory cytokines were evaluated by qRT‐PCR in colon samples (IFN-γ, TNF-α, IL-6, IL-10). DSS treatment caused a significant elevation of the levels of expression of these cytokines (**A-H**). Moreover, treatment with 16311 (**A-D**) or 145425 (**E-H**) did not modify gene expression when compared with their respective control mice/DSS-treated (**A-D** and **E-H** respectively). Data are shown as mean ± SEM. Kruskal‐Wallis test; *p < 0.05; **p < 0.01; ***p < 0.001.


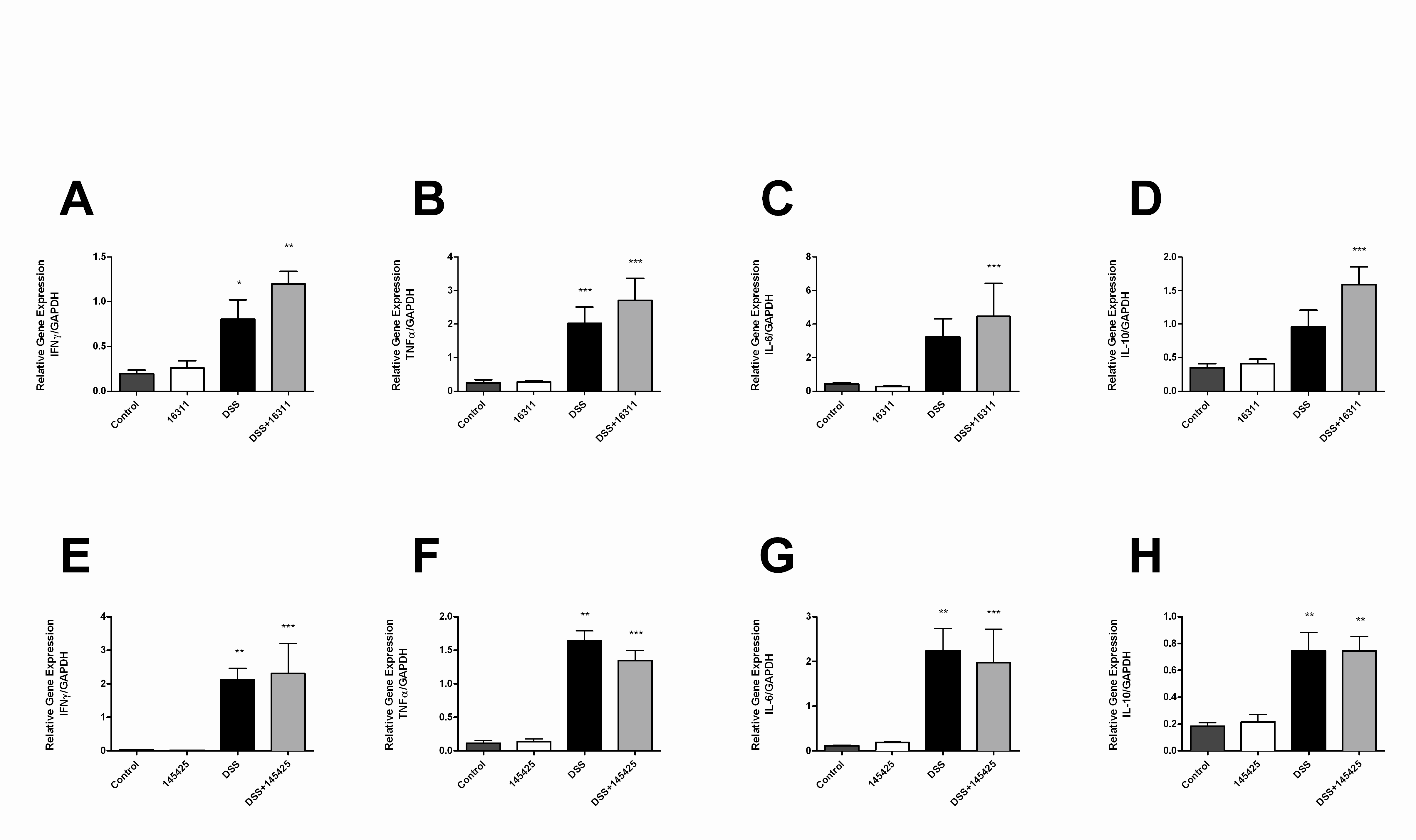


**Supplementary Figure S2. Adrenomedullin (AM) and adrenomedullin 2 (AM2) expression.** qRT-PCR results for AM (**A,C**) and AM2 (**B,D**) in colon samples treated with SM 16311 (**A,B**) or SM 145425 (**C,D**). SM 16311 significantly reduced the levels of AM in DSS-treated animals (**A**) but had no effect on AM2 expression (**B**). SM 145425 significantly increased AM expression in DSS-treated animals (**C**) and reduced AM2 expression in untreated mice (**D**). Data are shown as mean ± SEM. Kruskal‐Wallis test; *p < 0.05; **p < 0.01 versus untreated control. ^&^p < 0.05 versus DSS control.


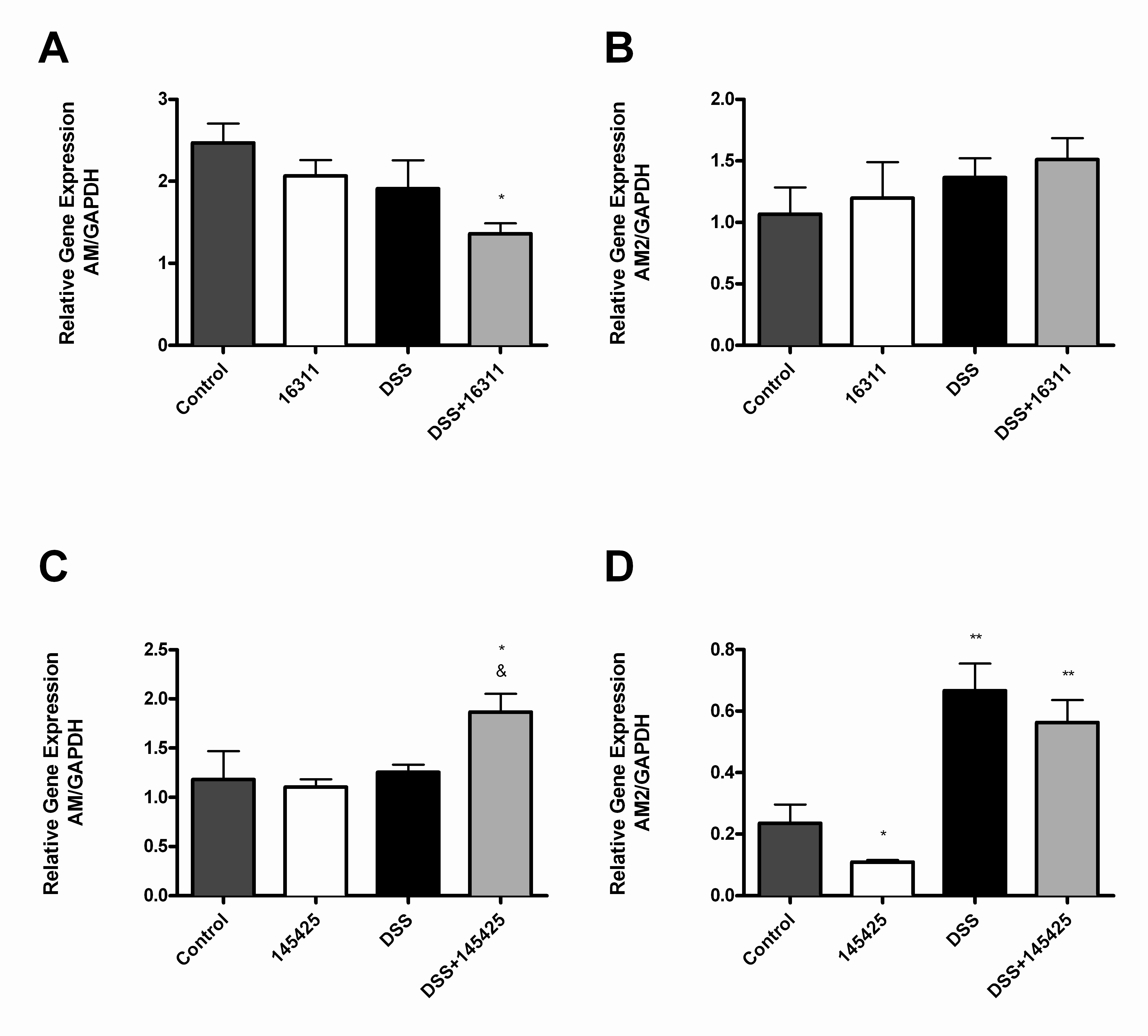


**Supplementary Figure S3. Myeloperoxidase activity**. Because the activity of the enzyme myeloperoxidase (MPO) is a useful indicator of the extent of neutrophil infiltration, the colon was homogenized and MPO activity in the supernatant was measured according to the manufacturer’s instructions (Cayman Chemical, Ann Arbor, MI). Mice treated with DSS showed drastically higher MPO activity compared with untreated mice, consistent with the severe intestinal inflammation observed in these animals. However, 145425 had no effect on this parameter. Data represent the mean ± SEM. Kruskal‐Wallis test; *p < 0.05; **p < 0.01.


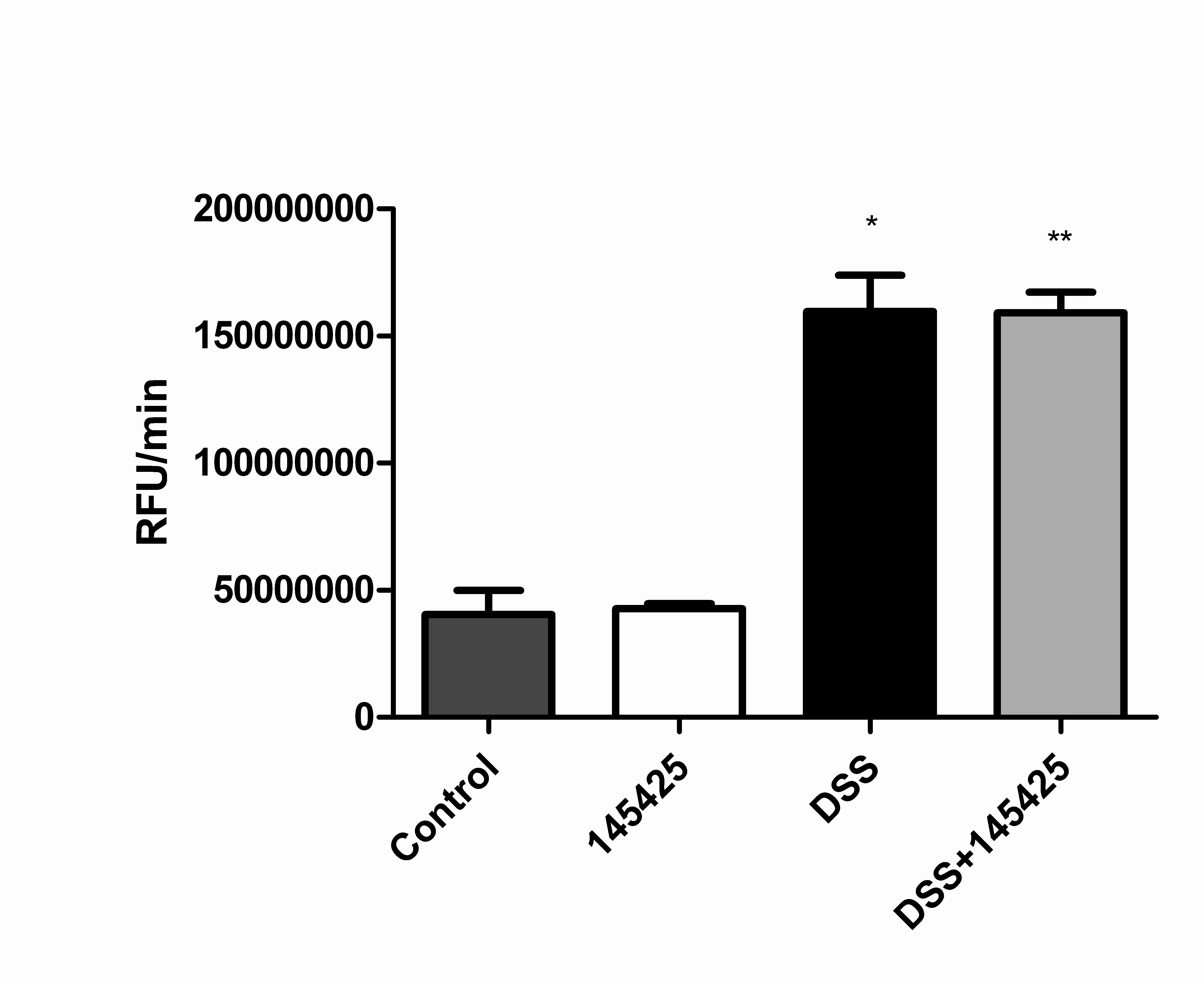


**Supplementary Figure S4. Analysis of gut bacterial communities by 16S rDNA sequencing.** SM 145425 administration modifies the gut microbiota. Percentages of the contributions of the indicated Phyla are shown. This graph was obtained from the OTU table using the phyloseq (R package). See also Table 2 for quantitative data.


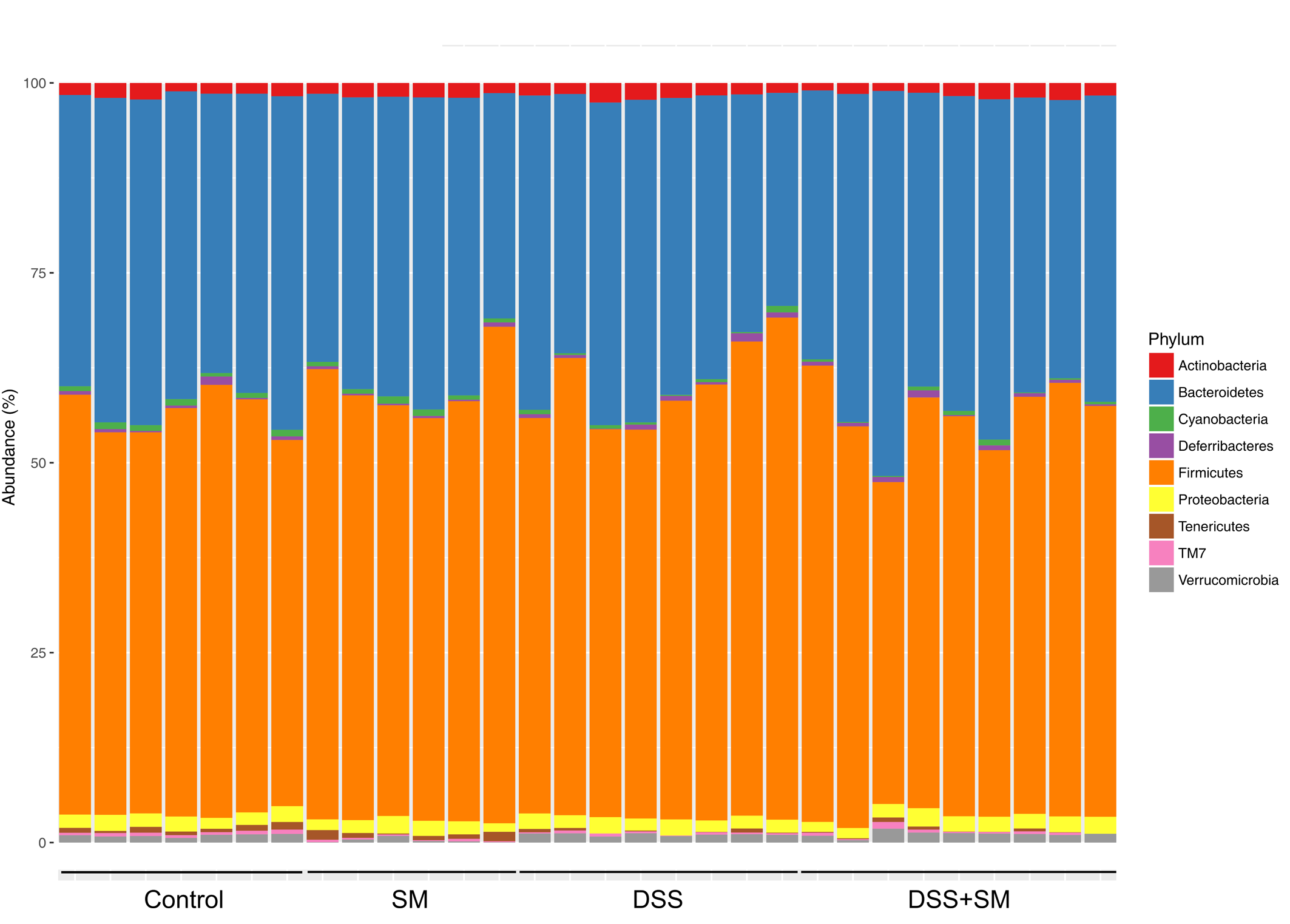


**Supplementary Figure S5. Alpha and beta-diversity.** The diversity of OTUs within a given sample is the alpha-diversity of that sample (**A**). The simplest way of measuring it is to enumerate OTUs present in that sample, also called species richness but other indexes such as Chao1 index, are also frequently used. Boxplots generated for the Chao1 alpha-diversity index are shown. Pairwise statistics are also generated by MicrobiomeAnalyst in a different output file (See Supplementary Table S5). The diversity of OTUs across samples is called beta-diversity (**B**). This is done by applying a distance metric over their taxonomic or genomic profiles that result in an all-against-all distance matrix. There are multiple ways to calculate distances between samples based on similarity of their members: one of the most common is the Bray-Curtis methodology that only considers the shared taxonomic composition across samples. Representative graphical output of the Beta-Diversity script (**B**). NMDS plot of generalized Unifrac distances showing distribution of the mouse fecal samples based on phylogenetic makeup of their microbiota. The plot shows a significant separation of sample groups according to groups (**B**).


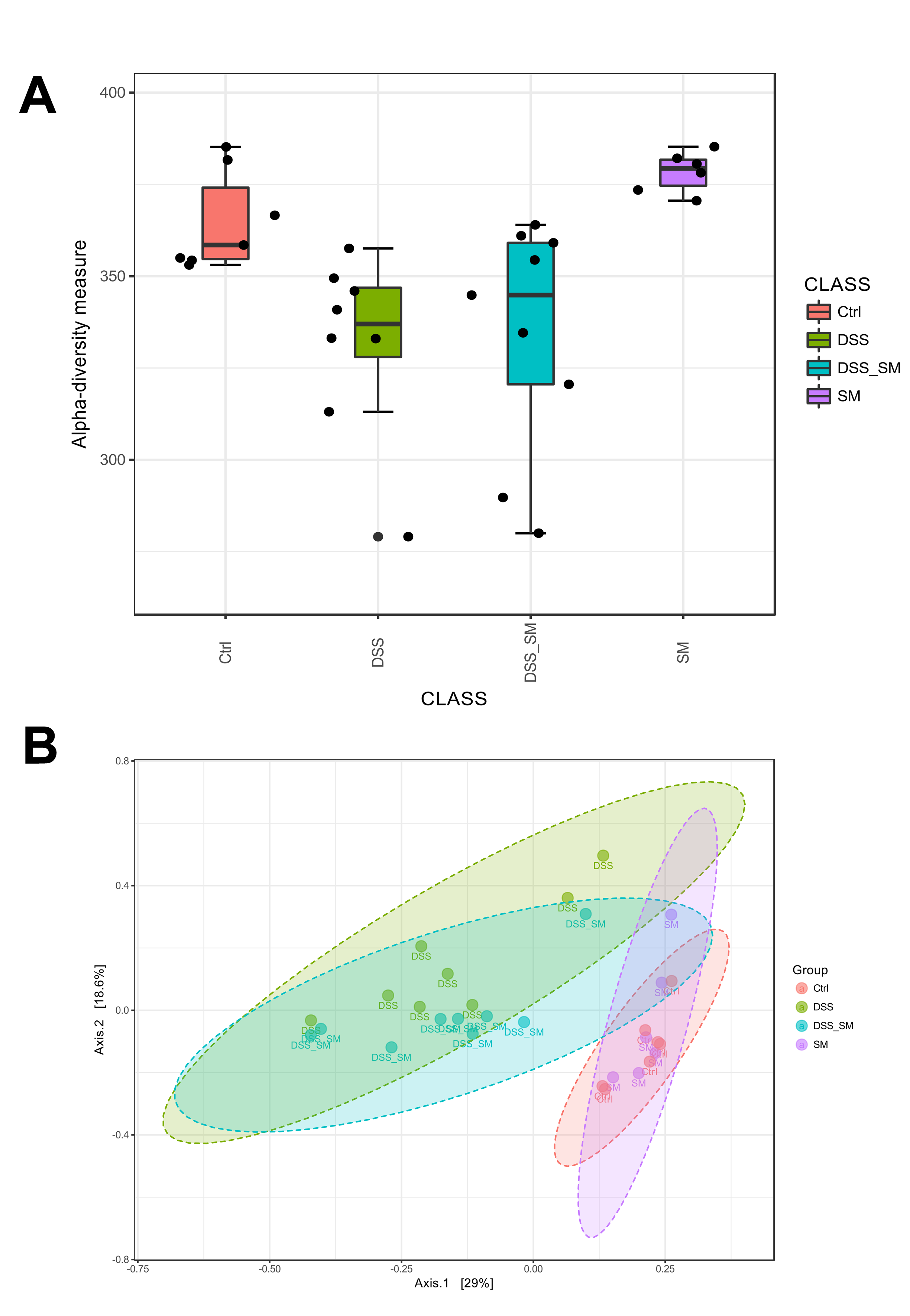


**Supplementary Table S1. Primer sequences used for quantitative real time PCR measurements.** The annealing temperature was 60ºC for all primers. GAPDH was used as housekeeping gene.

| **Name** | **Primer** |
| --- | --- |
| IFN-γ | Sense: 5'-ACTGGCAAAAGGATGGTGAC-3' |
|  | Antisense: 5'-TGAGCTCATTGAATGCTTGG-3' |
| TNF-α | Sense: 5'-ACGGCATGGA TCTCAAAGAC-3' |
|  | Antisense: 5'-AGATAGCAAATCGGCT GACG-3' |
| IL-6 | Sense: 5'-ATGGATGCTACCAAACTGGAT-3' |
|  | Antisense: 5'-TGAAGGACTCTGGCTTTGTCT-3' |
| IL-10 | Sense: 5'-AGGCGCTGTCATCGATTTCT-3' |
|  | Antisense: 5'-CTCTTCACCTGCTCCACTGC-3' |
| Lgr5 | Sense: 5'-CCTACTCGAAGACTTACCCAGT-3' |
|  | Antisense: 5'-GCATTGGGGTGAATGATAGCA-3’ |
| Erbb2 | Sense: 5'-GCTAGCGCGGCTTCTGAGAAA-3' |
|  | Antisense: 5'-ACCACAGGGTCTACCACTTCC-3' |
| AM | Sense: 5'-ATTGAACAGTCGGGCGAGTA-3' |
|  | Antisense: 5'-CTTGGTCTTGGGTTCCTCTG-3' |
| AM2 | Sense: 5'-GCTAGCAGATCCCAGCTTTG-3' |
|  | Antisense: 5'-GCAGGTAGAGGAGGCTGATG-3' |
| GAPDH | Sense: 5'-CATGTTCCAGTATGACTCCACTC-3' |
|  | Antisense: 5'-GGCCTCACCCCATTTGATGT-3' |

**Supplementary Table S2. Abundance of families which were significantly increased or decreased in feces of DSS mice compared to Control animals.**

| **Phylum** | **Taxonomic group** | **Variation** | **Statistical significance** |
| --- | --- | --- | --- |
| *Bacteroidetes* | *Bacteroidaceae* | Increased | * |
| *Firmicutes* | *Turicibacteraceae* | Increased | ** |
| *Firmicutes* | *Peptostreptococcaceae* | Increased | ** |
| *Firmicutes* | *Erysipelotrichaceae* | Increased | ** |
| *Proteobacteria* | *Alcaligenaceae* | Increased | * |
| *Bacteroidetes* | *Prevotellaceae* | Decreased | ** |
| *Bacteroidetes* | *Paraprevotellaceae* | Decreased | ** |
| *Cyanobacteria* | *c__4C0d-2;o__YS2* | Decreased | * |
| *Proteobacteria* | *Alphaproteobacteria;o__RF32* | Decreased | ** |
| *Tenericutes* | *Anaeroplasmataceae* | Decreased | * |

Kruskal‐Wallis test *p < 0.05; **p < 0.01.

**Supplementary Table S3. Abundance of lower taxonomic levels (family, and genus) which were significantly increased or decreased in feces from DSS+145425 mice compared to DSS** animals.

| **Phylum** | **Taxonomic group** | **Category** | **Variation** | **Statistical significance** |
| --- | --- | --- | --- | --- |
| *Bacteroidetes* | *Prevotellaceae* | Family | Increased | ** |
| *Bacteroidetes* | *Paraprevotellaceae* | Family | Increased | ** |
| *Bacteroidetes* | *Prevotella* | Genus | Increased | ** |

Kruskal‐Wallis test **p < 0.01.

***Supplementary Table S4. Abundance of lower taxonomic levels (family, and genus) which were significantly increased or decreased in feces from 145425 mice compared to Control animals.***

| **Phylum** | **Taxonomic group** | **Category** | **Variation** | **Statistical significance** |
| --- | --- | --- | --- | --- |
| *Firmicutes* | *Erysipelotrichaceae* | Family | Increased | ** |
| *Bacteroidetes* | *Paraprevotellaceae* | Family | Decreased | * |
| *Verrucomidales* | *Verrucomicrobiaceae* | Family | Decreased | ** |
| *Firmicutes* | *Allobaculum* | Genus | Increased | ** |
| *Bacteroidetes* | *Prevotella* | Genus | Decreased | * |
| *Verrucomidales* | *Akkermansia* | Genus | Decreased | ** |

Kruskal‐Wallis test *p < 0.05; **p < 0.01.

**Supplementary Table S5. Alpha-diversity statistics for each group. No differences were observed between Control (Ctrl) versus SM (145425) and DSS versus DSS+SM. However, differences were found between Control versus DSS groups.**

| **Group1** | **Group2** | **Group1 mean** | **Group1 std** | **Group2 mean** | **Group2 std** | **t stat** | **p-value** |
| --- | --- | --- | --- | --- | --- | --- | --- |
| **DSS** | **SM** | 318.8152 | 21.9814 | 367.1989 | 11.1702 | -4.5687 | 0.0038 |
| **DSS** | **DSS+SM** | 318.8152 | 21.9814 | 316.1074 | 35.9137 | 0.1735 | 0.8645 |
| **Ctrl** | **DSS+SM** | 351.0307 | 13.4823 | 316.1074 | 35.9137 | 2.2846 | 0.0576 |
| **Ctrl** | **DSS** | 351.0307 | 13.4823 | 318.8152 | 21.9814 | 3.1310 | 0.0159 |
| **SM** | **DSS+SM** | 367.1989 | 11.1702 | 316.1074 | 35.9137 | 3.1442 | 0.0232 |
| **Ctrl** | **SM** | 351.0307 | 13.4823 | 367.1989 | 11.1702 | -2.1439 | 0.0662 |
